# Une mucormycose faciale compliquant une angiocholite grave: à propos d’un cas

**DOI:** 10.11604/pamj.2016.25.246.10977

**Published:** 2016-12-21

**Authors:** Karim Lakhdar, Naoufal Houari, Abderrahim Elbouazzaoui, Taoufik Ameuraoui, Brahim Boukatta, Hicham Sbai, Nabil Kanjaa

**Affiliations:** 1 Service de Réanimation Polyvalente A4, CHU Hassan II, Fès, Maroc; 2 Service de Radiologie CHU Hassan II, Fès, Maroc

**Keywords:** Mucormycose, opportuniste, angiocholite grave, Mucormycosis, opportunistic, severe angiocholitis

## Abstract

Les mucormycoses sont des infections fongiques opportunistes survenant chez des patients immunodéprimés. C’est une affection grave compromettant le pronostic vital. Même diagnostiquée précocement, la mortalité des mucormycoses atteint 50%. Nous rapportons le cas d’une mucormycose chez une patiente diabétique hospitalisée en réanimation pour angiocholite grave. L’évolution était fatale.

## Abstract (en)

Mucormycosis is an opportunistic fungal infection occurring in immunocompromised patients. It is a serious life threatening condition. Even though diagnosed early, mucormycosis has a mortality rate reaching 50%. We report the case of mucormycosis in a diabetic patient hospitalized in Intensive Care Unit for severe angiocholitis. Evolution was lethal.

## Introduction

Les mucormycoses sont des infections fongiques opportunistes rares, survenant chez des patients immunodéprimés. Elles sont dues à des champignons filamenteux appartenant à la classe des zygomycètes de type mucoral. Les mécanismes physiopathologiques à l’origine de la survenue de cette mycose sont mal connus. Les localisations de mucormycose décrites sont rhino-cérébrale, pulmonaire, cutanée, digestive, cérébrale et disséminée. Nous rapportons le cas d’une mucormycose survenant chez une patiente diabétique hospitalisée en réanimation pour angiocholite grave d’origine lithiasique; le diagnostic a été retenu sur des critères cliniques et mycologiques.

## Patient et observation

Nous rapportons l’observation de Mme M.H, âgée de 37 ans, diabétique depuis 5 ans sous antidiabétiques oraux, cholécystectomisée il y’a 4 ans, admise aux urgences dans un tableau d’ictère fébrile d’évolution aigue depuis 3 jours. L’examen à son admission a trouvé une patiente confuse, ictérique, fébrile à 39,1°, tachycarde à 120 bpm avec une pression artérielle à 100/56 mm-Hg, une glycémie capillaire à 4,5 g/l et une acétonurie positive. La patiente a été admise en salle de déchoquage, où elle a bénéficié d’une mise en position demi-assise, un monitorage de la pression artérielle non invasive, de la saturation périphérique en oxygène, du tracé électro-cardiographique, avec mise en place de deux voies veineuses de calibre 16 gauges. La patiente a bénéficié d’une oxygénothérapie nasale à raison de 4 litres par minute et d’une réhydratation intraveineuse par des cristalloïdes avec correction de la décompensation acidocétosique. La patiente a bénéficié d’une échographie hépatobiliaire objectivant une dilatation des voies biliaires intra-hépatiques proximales avec importante dilatation de la voie biliaire principale à 30mm en amont de multiples calculs du bas cholédoque. Le bilan biologique a objectivé une hémoglobine (Hb) à 13,3 g/dL, un taux de plaquettes (Plq) à 397000 éléments/mm^3^, une hyperleucocytose à 27300 éléments/mm^3^ avec une protéine C réactive (CRP) à 371, une fonction rénale normale et un bilan de cholestase perturbé. La patiente a été acheminée en salle de cathétérisme avec réalisation d’une cholangiographie rétrograde par voie endoscopique pour évacuation des calculs puis a été acheminée en réanimation pour surveillance et complément de prise en charge. La patiente a été mise sous réhydratation intraveineuse, ration de base, antibiothérapie à base de ceftriaxone et métronidazole, protection gastrique, anti-coagulation préventive avec insulinothérapie en fonction de la glycémie capillaire. L’évolution a été marquée par une amélioration clinique avec amélioration de l’état de conscience, régression de la fièvre et de la tachycardie avec normalisation du bilan biologique.

**Figure 1:**
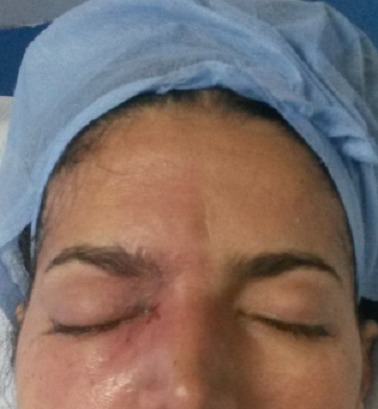
Œdème facial avec apparition d'une plaque érythémateuse périorbitaire droite

**Figure 2:**
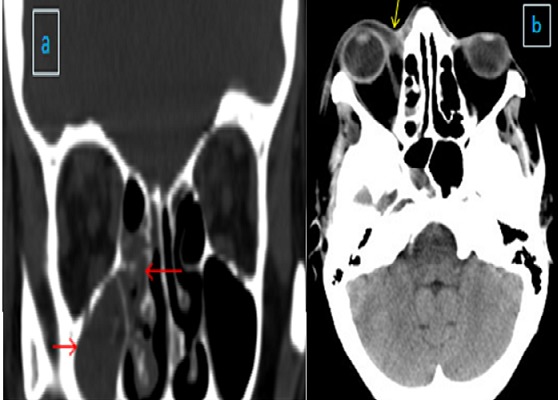
TDM orbito-faciale: A) coronale; B) axiale: importante infiltration des parties molles palpébrales gauches associée à un comblement du sinus maxillaire et des cellules ethmoïdales homolatérales

**Figure 3:**
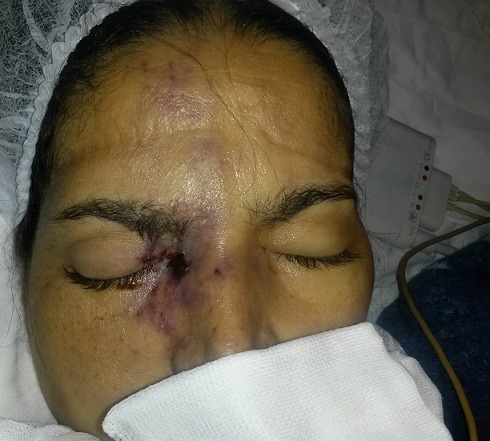
Extension de l'œdème facial avec aggravation de la lésion érythémateuse et apparition d'une nécrose en son centre

**Figure 4:**
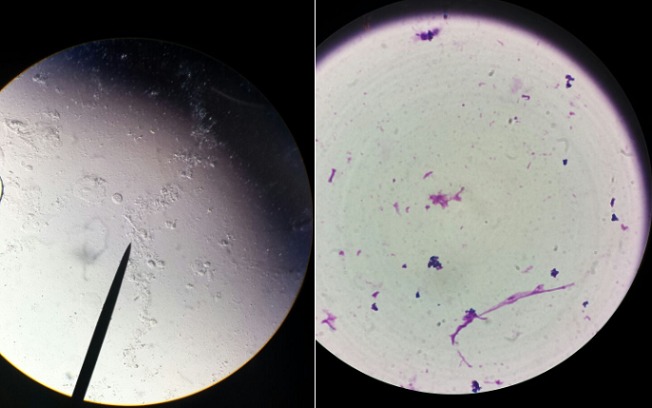
Filament mycélien allongé en microscopie directe (flèche)

**Figure 5:**
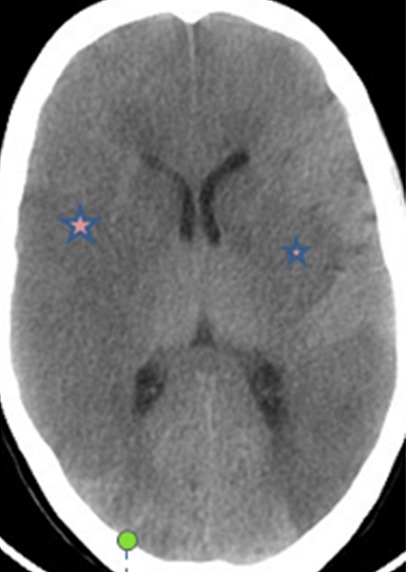
TDM cérébrale (coupe axiale): AVC ischémique carotidien bilatéral aigu suite à une thrombose inflammatoire

Au 3^ème^ jour d’hospitalisation, la patiente a présenté une légère confusion, un œdème palpébral bilatéral, une plaque érythémateuse périorbitaire droite (Figure 1), des céphalées intenses avec un plateau fébrile à 39°. Le bilan biologique a objectivé une hyperleucocytose à 19000 éléments/mm^3^ avec une CRP à 215. Un bilan infectieux fait d’hémocultures (HC) et d’examen cytobactériologique des urines (ECBU) ainsi qu’un prélèvement mycologique au niveau de la lésion périorbitaire a été réalisé. Une TDM orbito-faciale a été réalisée objectivant une importante infiltration des parties molles palpébrales droites associée à un comblement du sinus maxillaire et des cellules éthmoïdales homolatérales (Figure 2). L’évolution au 4^ème^ jour d’hospitalisation a été marquée par l’extension de la plaque érythémateuse périorbitaire droite avec début de nécrose cutanée (Figure 3), aggravation de l’œdème facial ainsi qu’une aggravation neurologique avec un score de Glasgow devenant à 8 et une aggravation hémodynamique avec chute tensionnelle et oligurie ne répondant pas au remplissage vasculaire d’où l’intubation-ventilation de la patiente sur des critères neurologiques et hémodynamiques. La patiente a été sédatée et mise sous noradrénaline à une dose de 0,4 microg/kg/min. Le prélèvement mycologique a mis en évidence des filaments mycéliens de type mucoral (Figure 4) d’où la mise de la patiente sous voriconazole à une dose de 800mg puis 200 mg/12h par manque d’amphotéricine B pour suspicion de mucormycose orbito-faciale. Une TDM cérébrale a été réalisée objectivant un accident vasculaire cérébral carotidien bilatéral aigu suite à une thrombose inflammatoire (Figure 5). Les suites ont été marquées par l’installation d’une défaillance multiviscérale avec décès de la patiente au 5^ème^ jour d’hospitalisation suite à un état de choc septique réfractaire.

## Discussion

Les mucormycoses sont des infections à champignons filamenteux de l’ordre des Mucorales, appartenant à la famille des Zygomycètes. Les genres les plus fréquemment retrouvés sont le Rhizopus, Absidia et Mucor [1]. C’est une affection retrouvée généralement chez des patients immunodéprimés ou en décompensation acidocétosique. L’affection est opportuniste et survient chez les patients atteints d’hémopathie maligne, les porteurs d’insuffisance rénale chronique, après transplantation d’organe ou en particulier chez des patients sous traitement par Déféroxamine. En effet, cet agent chélateur de fer stimule la croissance de certaines mucorales (Rhizopus microspores et Rhizopus arrizhus) et augmente leur pathogénécité [2]. Pour les hémopathies malignes, les mucormycoses surviennent principalement chez les patients atteints de leucémies aigues et de lymphomes avec une neutropénie profonde [3]. Dans notre contexte, le diabète en décompensation acidocétosique ainsi que le sepsis secondaire à une angiocholite grave sont les principaux facteurs de risque. Les mucorales se caractérisent par un tropisme vasculaire très important expliquant ainsi leur pouvoir de dissémination hématogène et leur aptitude à entraîner des nécroses ischémiques dans les tissus infectés [4]. La forme rhino-orbito-cérébrale représente 40 à 75% des mucormycoses [5]. La symptomatologie observée dans cette forme clinique est faite de : œdème facial, obstruction nasale, douleur faciale, rhinorrhée, fièvre, chémosis et une destruction palatine [3]. La symptomatologie chez notre patiente était dominée par un état fébrile et une cellulite orbito-faciale. Elle s’est généralisée par la suite par des signes neurologiques faits de troubles de conscience avec des signes hémodynamiques à travers le collapsus cardiovasculaire. Le diagnostic est basé sur des critères mycologiques et anatomo-pathologiques. Les prélèvements mycologiques montrent des filaments mycéliens larges avec un angiotropisme entrainant la formation de thromboses septiques et d’infarctus tissulaire. Seule la culture permet l’identification de l’espèce [5]. Le traitement d’une mucormycose chez un patient immunodéprimé repose sur une chirurgie agressive destinée à stériliser les tissus atteints et sur la prescription d’un traitement antifongique [3]. Si un débridement chirurgical large ne peut être réalisé, l’amphotéricine B en monothérapie peut être utilisée [6]. Non traitée, l’évolution de cette infection est fatale. Même diagnostiquée précocement, la guérison n’est obtenue que dans la moitié des cas [2].

## Conclusion

La mucormycose est une affection rare, souvent mortelle, touchant généralement des patients immunodéprimés. Dès que le diagnostic est évoqué, un traitement adapté doit rapidement être instauré afin de diminuer la morbi-mortalité liée à cette pathologie.
